# HTS-Based Monitoring of the Efficiency of Somatic Embryogenesis and Meristem Cultures Used for Virus Elimination in Grapevine

**DOI:** 10.3390/plants9121782

**Published:** 2020-12-16

**Authors:** Mihaly Turcsan, Emese Demian, Tunde Varga, Nikoletta Jaksa-Czotter, Erno Szegedi, Robert Olah, Eva Varallyay

**Affiliations:** 1 Research Institute for Viticulture and Oenology, Experimental Station of Kecskemet, National Research and Innovation Center, Katona Zsigmond Street 5, 6000 Kecskemet, Hungary; turcsan.mihaly@szbki.naik.hu (M.T.); szehome@t-online.hu (E.S.); olah.robert@szbki.naik.hu (R.O.); 2 Agricultural Biotechnology Research Institute, National Research and Innovation Center, Szent-Gyorgyi Albert Street 4, 2100 Godollo, Hungary; demian.emese@abc.naik.hu (E.D.); varga.tunde07@gmail.com (T.V.); czotter.nikoletta@abc.naik.hu (N.J.-C.)

**Keywords:** somatic embryogenesis, meristem culture, virus elimination, sRNA HTS

## Abstract

Meristem culture and somatic embryogenesis are effective tools for virus elimination of vegetatively propagated crops including grapevine (*Vitis vinifera* L.). While both have been shown to be useful to eliminate the main grapevine viruses, their efficiency differs depending on the virus and grapevine variety. In our work, we investigated the efficiency of these two virus elimination methods using small RNA high-throughput sequencing (HTS) and RT-PCR as virus diagnostics. Field grown mother plants of four clones representing three cultivars, infected with different viruses and viroids, were selected for elimination via somatic embryogenesis (SE) and meristem culture (ME). Our results show for the first time that using SE, elimination in mother plants was effective for all viruses, i.e., grapevine rupestris vein feathering virus (GRVFV), grapevine Syrah virus 1 (GSyV-1), Grapevine virus T (GVT) and grapevine Pinot gris virus (GPGV). This study also confirms previous studies showing that SE is a possible strategy for the elimination of GFkV, GRSPaV, HSVd, and GYSVd-1. Our results demonstrate that the efficacy of virus elimination via SE is relatively high while the purging of viroids is lower. Our work provides evidence that the efficiency of SE is comparable to that of the technically difficult ME technique, and that SE will offer a more effective strategy for the production of virus-free grapevine in the future.

## 1. Introduction

Vineyards represent investments that often persist for many decades. To ensure their health, it is essential to use high quality propagating material (rootstocks and scions), free from pathogens, during their establishment. The lack of efficient methods to produce and verify clean planting stock is especially important in case of phytoplasmas and viruses. The elimination of phytoplasmas may be accomplished by a simple hot water treatment of the canes, while virus elimination requires more complex and specialized methods that may vary among different pathogen species. More than 80 viruses and many viroids can infest grapevine, including some that cause important and economically-significant diseases [1], and recently described ones whose symptoms are yet to be fully determined. In case of viruses, meristem culture (ME)-based elimination relies on the fact that viruses are usually excluded from the meristematic zone of the shoot tips [2]. *Vitis vinifera* is a recalcitrant species for in vitro culture, and different cultivars exhibit different responses [3]. The efficiency of shoot regeneration is also dependent on the size of the isolated meristem [4,5]. The maintenance of a virus-free status mandates the culture of extremely small shoot tip meristems, rendering the process technically challenging [6].

Somatic embryogenesis (SE) is an alternative method to overcome these challenges [7,8], and has been shown to eliminate viruses and viroids in other vegetatively-propagated crop species such as cassava [9], cocoa [10], Citrus [11,12] and sugarcane [13]. The first results for SE based elimination of viruses from grapevine were published by Goussard and coworkers [14], where grapevine leafroll associated viruses (GLRaV-2 and -3) were eliminated from ‘Roobernet’ cultivar. Using this method, several other viruses and even viroids were successfully eliminated from different cultivars [15,16,17,18,19,20,21,22,23,24]. SE protocols vary according to the tissue explant, growth regulator type and concentration, and the direct or indirect somatic embryogenesis strategy (see Table S1 for details and references).

The efficiency of the virus elimination is generally monitored by virus diagnostic methods, mostly by DAS-ELISA, RT-PCR, RT-qPCR alone or in different combinations (see Table S1 for details). Serological and PCR methods to detect viruses are reliable and sensitive, but are specific to single pathogens or strains. Although these analytical methods are effective for monitoring the virus elimination process, they are not sufficient to verify the virus-free status of host cultivars. As an alternative, small RNA HTS can be used to detect all of the pathogens present in the sample [25].

Both ME and SE may be used effectively to eliminate most well-studied viruses and viroids from grapevine. Since grapevine may be infested simultaneously with many different viruses, different protocols for their elimination may be necessary. The appearance of new, destructive viruses on grape has presented additional challenges for the production of clean planting stocks. We report results herein on small RNA HTS to investigate the viromes of four grapevine cultivars and for monitoring virus elimination by SE and ME.

## 2. Results

### 2.1. Virus Diagnostics of the Mother Plants

Four field-grown mother plants of three cultivars, two clones of ’Muscat Ottonel’ (MO7 and MO14), ‘Trilla’, and ‘Sziren’ (new breeds at the grapevine collection of National Agricultural Research and Innovation Center, Research Institute for Viticulture and Oenology, Experimental Station of Kecskemet), were selected for virus elimination. Virus detection in these four mother plants was done via small RNA HTS (see Table 1 and Tables S2–S4 for details) and validated by RT-PCR as an unbiased method (see Figure S1).

The mother plants were infected with four to six viruses, including GFkV, GRVFV, GSyV-1, GRSPaV, GVT, and GPGV, as well as two viroids, HSVd and GYSVd-1. The results of the small RNA HTS and RT-PCR were very similar for GFkV, GRVFV, GSyV-1, and GPGV, as well as for the viroids (Table 2).

In contrast, and in agreement with our previous observations, small RNA HTS failed to detect GRSPaV infection [26,27]; we observed the same contradictions for GVT. GVT is a close relative of GRSPaV, and we hypothesize that its detection could have failed because of (i) its high variability or (ii) its low level of produced small RNA. Inflorescences were the explants for SE, while for ME, in vitro cultures of these mother plants were the explant tissues.

### 2.2. Virus Elimination Using Somatic Embryogenesis

Embryogenic callus cultures from all four mother plants were established with different success rate, depending on the genotype (see Table 3 for the results). Somatic embryo development and plant regeneration were successfully induced from all four genotypes.

### 2.3. Virus Elimination Using a Meristem Culture

In vitro cultures of the ’Muscat Ottonel’ clones were very difficult to establish. We could not regenerate a sufficient number of meristems because of the very poor in vitro plant development and limited shoot numbers under the conditions that we used (see Table 4 for results). As elimination failed for this cultivar, we were able to compare the efficiency of ME with SE only in the case of ‘Trilla’ and ‘Sziren’.

### 2.4. Comparison of the Efficiency of the Sanitation Methods

The efficiency of virus elimination was controlled by small RNA HTS as a virus diagnostic method. Pools prepared from the individual sanitized lines were analyzed via small RNA HTS (see Table 1 for the Library codes and Tables S2 and S3 for detailed information). The result was validated with RT-PCR (Table 5, Tables S3 and S4, and Figure S1). When inefficient elimination for a particular virus was observed, the independent lines were individually tested by RT-PCR to determine whether any of those lines were virus free (Figures S2–S5).

GFkV and GRVFV were present only in the Muscat Ottonel clones, and were successfully eliminated by SE. GSyV-1 was present in all of the mother plants, and this virus was eliminated at 100% efficiency using both SE and ME. GRSPaV was also present in all of the mother plants. Its detection with small RNA HTS did not coincide with the RT-PCR results (Table 5). For the RT-PCR test of the pools, we used two sets of primers, but for the individual lines, we used only the most sensitive primers (Figures S1–S5). As a result, we found that SE was successful in elimination of GRSPaV. For MO7, MO14, and ‘Trilla’, the efficiency was 100%, while for ‘Sziren’, it was only 54%. As with these sensitive primers, we detected the virus in 6 of the 11 virus eliminated independent lines. However, SE was more efficient than ME; with SE, only 1 out of 5 (20% in the case of ‘Trilla’) and 2 out of 4 (50% in the case of ‘Sziren’) virus eliminated lines was GRSPaV-free (Table 5 and Figures S2–S5). GVT was present in three of the mother plants according to the RT-PCR results, while small RNA HTS was only detected it in ‘Sziren’ ME lines. To check if the virus eliminated lines were free from this virus, all of the individual lines were tested by RT-PCR and showed that GVT was successfully eliminated by SE from all of the plants. However, using ME, the efficiency was lower, with 4 out of 5 (80% in the case of ‘Trilla’) and 2 out of 4 (50% in the case of ‘Sziren’) lines were GVT-free (Table 5 and Figures S2–S5). GPGV was present in three mother plants; by using SE, this virus was successfully eliminated from all of the plants. SE was more efficient for the elimination of this virus than ME, as only 3 out of 5 (60% for ‘Trilla’) and 2 out of 4 (50% for ‘Sziren’) lines were GPGV-free after ME (Table 5 and Figures S4 and S5).

HSVd and GYSVd are the most widespread viroids in grapevine. Both were present in all four mother plants. Their elimination was not always successful, and the small RNA HTS and RT-PCR gave different results in some cases (Table 5). Systematic virus diagnostics of the independent lines showed that this contradiction appeared when not all, but only some, of the lines were infected with this viroid. HSVd was successfully eliminated with SE in 100% of the MO7 and MO14 lines. For ‘Trilla’ and ‘Sziren’, this rate was a bit lower. HSVd was found in only 2 out of 12 and 2 out of 11 lines, indicating 83% and 81% efficiency, respectively (Table 5 and Figures S2–S5). GYSVd elimination was slightly less efficient. Using SE, 6 out of 7 (for MO7), 6 out of 8 (for MO14), and 9 out of 11 (for ‘Sziren’) lines were successfully eliminated, but for ‘Trilla’, this ratio was only 1 out of 12. In ‘Trilla’, we could not obtain a GYSVd-free line with ME, while all 5 lines of ‘Sziren’, prepared with the same method, were free from this viroid (Table 5 and Figures S2–S5).

## 3. Discussion

In our work, we used SE and ME for grapevine and monitored the virus elimination efficiency using small RNA HTS and RT-PCR in parallel as a diagnostic method. It is already known that the efficiency of virus elimination protocols can differ among varieties. We found that both SE and ME had 100% efficiency in eliminating GFkV, GRVFV, and GSyV-1 from plants; their efficiency was also very good against GVT and GPGV. To the best of our knowledge, there is no data available on GRVFV, GSyV-1, GVT, and GPGV being eliminated through somatic embryogenesis. Some grapevine cultivars infected with GPGV were successfully sanitized by the meristem culture with or without heat therapy [28], or by repeated treatment with ribavirin for 8 to 16 weeks [29], but we are not aware any reported for elimination of GRVFV, GSyV-1 and GVT. It is challenging to eliminate GRSPaV [5], and traditional virus elimination methods showed very variable efficiency [21,30]. As an alternative, chemotherapy was tested and proven to be efficient; however, that protocol also needs optimization to avoid the high cytotoxic effect of the used reagents [5]. In our work, we found that SE could be a very good alternative to eradicate GRSPaV from grapevine, as most of the regenerated lines were free from this virus. The elimination of viroids is always a crucial factor. Somatic embryogenesis proved to be useful for both HSVd and GYSVd [20]. Moreover, by using in situ hybridization, rearrangement of the viroids in the calli were demonstrated [20]. Thermotherapy did not efficiently eliminate viroids from the mother plants [20], but they could be efficiently eliminated via meristem tip cultures. The use of meristem tip cultures is a very difficult technique because the regeneration efficiency of the prepared shoot tips is higher when the shoots are longer (˃0.5 mm); however, this added length also adversely affects the virus level [31]. We were only able to regenerate ME lines from the two cultivars of ‘Trilla’ and ‘Sziren’ but not from ‘Muscat Ottonel’ plants. Small RNA HTS-based virus diagnostics showed that the efficiency of this method was inferior to that of SE. When we tested the resulting individual lines for the presence of these viruses and viroids, we found that most of the viruses and viroids that were present in the mother plant were still present in two of the lines that were regenerated from ‘Sziren’, possibly because, in these cases, longer portions of the apical shoots still containing the pathogens were used for the meristem culture, highlighting the importance of this technical factor. Our work demonstrates that SE is a very efficient alternative to ME for grapevine virus elimination, but the stability of the regenerated lines and their constant virus free status have to be further monitored in the following years, which is currently in progress.

## 4. Materials and Methods 

### 4.1. Plant Material

The selected mother plant shoots (including the shoot tips, young leaves, older leaves, inflorescences, and tendrils) were collected in May 2017. In vitro cultures were sampled after virus elimination. Whole plants of in vitro growing vines, representing independent lines, were used for virus diagnostics. RNA was extracted from the sampled plant materials via the CTAB method [32]. RNA pools representing individual plants or independent regenerated lines were prepared by mixing equal amounts of RNA from different organs (for the mother plants) and from the sampled individual lines (for the sanitized in vitro plants). Small RNA sequencing libraries were prepared from the purified small RNAs using an TruSeq Small RNA Library Preparation Kit (Illumina) and our in-house-modified protocol [33]. Ten small RNA libraries (four from the mother plants and six from the sanitized cultivars) were prepared (see Table 1 for the library codes and Table S2 for details) and sequenced using a single index on a HiScanSQ by UD-Genomed (Debrecen, Hungary) (50 bp single-end sequencing, with 8 samples/sequencing lane). The FASTQ files of the sequenced libraries have been deposited in the GEO (GSE159758).

### 4.2. Grapevine In Vitro Cultures

Shoot tips and inflorescences were surface-sterilized by submersion in ethyl alcohol (70%) for 30 s, followed by rinsing in a sodium hypochlorite solution (ca. 0.6% NaOCl, 0.1% Tween 20) for 10 min; then, the samples were washed three times in sterile distilled water.

The medium prepared for the in vitro cultures contained Murashige and Skoog macroelements in half concentration; MS microelements and vitamins in full concentration; and folic acid (0.002 mg/L), biotin (0.002 mg/L), calcium-pantothenate (0.4 mg/L), p-aminobensoic acid (0.2 mg/L), riboflavin (0.2 mg/L), L-arginine (10 mg/L), and L-glutamine (100 mg/L) as a supplement. The pH was adjusted to 5.80 with 3% (*w*/*v*) KOH. The plant materials were transferred monthly to a fresh medium.

### 4.3. Meristem Cultures

To establish the in vitro cultures, hot-water treated (51 °C for 30 min) wooden canes were cut into single budded nodes and rooted in granulated perlite moistened with tap water. Then, 0.5–1 cm-long tips of the shoots formed by these buds were surface-sterilized and placed on the above-mentioned basal medium containing 30 g/L sucrose and 0.5 mg/L meta-topolin (mT). The developing shoots were micro-propagated on the same medium without hormone.

Meristems of the in vitro plants (with sizes of 0.2–0.5 mm) were carefully excised under a microscope and placed on a medium in Petri dishes including 30 g/L sucrose and 1 mg/L mT and solidified with 6 g/L agar. Meristems were kept in the dark for three days by wrapping the Petri dishes in aluminium foil to promote shoot formation and elongation rather than cotyledon or leaf growing [34].

Developing plantlets without roots were passed into the medium in vials with the same media supplemented with 30 g/L sucrose, 0.2 mg/L mT and 6 g/L agar.

Well-developed individual meristematic lines with roots were micro-propagated on the same hormone-free medium in jars supplemented with 10 g/L sucrose and 3 g/L gelrite. Plantlets showing poor growth or yellowing were first transferred to the MSAc medium [35] before the micropropagation on the aforementioned medium.

### 4.4. Somatic Embryogenesis

SE was carried out according to the previously described method [36]. Briefly, inflorescences of the four mother plants were collected at developmental stage 5 [37]. After surface sterilization, the anthers with their filaments attached were excised and placed on a solid MST medium. The MST medium was prepared by supplementing the basal medium with 0.05 mg/L TDZ, 1.1 mg/L 2,4-D, and 20 g/L sucrose, which were solidified with 5 g/L agar (20573-0-33, REANAL). The anther cultures were incubated at 24 °C in the dark. 

Calli with embryogenic morphologies were passed into the basal medium supplemented with 1 g/L activated charcoal (MSAc), 10 g/L sucrose, and 3 g/L gelrite (Duchefa). The same medium was used during embryo development, germination, plant regeneration, and rooting.

Individually regenerated plants were micro propagated on the same medium without activated charcoal.

### 4.5. Pipeline for Data Evaluation of the HTS Results (Bioinformatics)

We used the CLC Genomics Workbench (Qiagen) for bioinformatics analysis. After trimming and quality control, the reads were used for *de novo* assembly to build longer contigs from the non-redundant reads by employing an assembler of CLC (*de novo* assembly) using the default options: word size 20, bubble size 50, and simple contig sequences with a minimum 35nt length (see Table S3 for initial statistics of the analysis). Annotation of these contigs was performed using the blastn algorithm with the default options (thread 1, word size 11, match 2, mismatch 3, gap cost existence 5, and extension 2) and the NCBI plant-hosted viral reference genomes (downloaded at 0307 2019). When viruses were detected (i.e., when at least one virus specific contig was present), the reads were directly mapped to the reference genome and were counted with and without redundancy (using the map to the reference command allowing 1 mismatch). The number of normalized reads (read/1 million reads—RPM) was then calculated from the mapped redundant reads and the number of total sequenced reads. Based on this mapping, a consensus sequence was prepared and used to calculate the coverage (%) of the viral genome. If at least two parameters from any of the following were fulfilled, we further investigated the presence of the given virus or viroid by RT-PCR, as an independent virus diagnostic method: (i) the presence of any virus specific contigs, (ii) a number of normalized redundant virus specific reads >200, or (iii) coverage of the virus genome >60%/coverage of the viroid genome >80%.

### 4.6. Validation of Predicted Viral Diagnostics by RT-PCR

Pooled RNA extracts representing each mother plant and pools of the independent lines prepared with the specified method was used as templates for cDNA synthesis with a RevertAid First Strand cDNA Synthesis Kit (Thermo Fisher Scientific, USA) using random primers according to the manufacturer’s instructions. cDNA from all of the independent in vitro lines was also generated by the same method. The generated cDNA was used for the PCR analysis (the sequences and important parameters of the primers used to amplify the viral parts, together with the applied annealing temperatures, are provided in Table S5) performed with Phire Hot Start II DNA Polymerase (Thermo Fisher Scientific). The quality of the cDNA was tested by amplifying a part of the *V. vinifera* actin gene. The result of the PCR was evaluated via gel electrophoresis of the PCR products.

## 5. Conclusions

Vineyards that have been planted for decades are continuously endangered by several viruses and viroids. The best way to keep the infection risk low is prevention, which mainly involves the usage of virus-, viroid-, and phytoplasma-free propagation materials. To maintain the valuable traits of the cultivars, grapevine is propagated vegetatively. To prevent the distribution of viruses, virus diagnostics of the mother plants at the certified virus-free vineyards have key importance. Mother plants of the new breeds must be proven to be virus free, and, if not, must be sanitized. In our work, in line with the previous results, we demonstrated that the SE method is efficient not only for eliminating most viruses, but also for eliminating viroids [20]. The artificial infection of grapevines with particular viruses for virus indexing is very difficult and time-consuming, which is why systematic tests of the efficiency of virus elimination methods are highly demanding. The advantages of the meristem culture can be combined with chemotherapy, thereby helping to overcome these technical challenges [29], but these methods are not only dependent on the virus, but also on the genotype of the host plant, which makes this picture more complex. Virus elimination with somatic embryogenesis offer very useful possibilities. Moreover, SE seems to be a very good alternative for the more technically challenging ME. However, during SE, somaclonal variation may occur. With our protocol, the regeneration rate was very high for all of the investigated cultivars, but the genetic stability of the SE-sanitized grapevines must be further verified. We must also confirm that none of the cultivar-specific properties was affected.

## Figures and Tables

**Table 1:** Summary of the prepared small RNA HTS sequencing libraries.

Cultivar/Clone	Status of the Plant	Library Code
**Muscat Ottonel 7**	**Mother Plant**	1_MO7
**Muscat Ottonel 14**		2_MO14
**Trilla**		3_T
**Sziren**		4_SZ
**Muscat Ottonel 7**	**Lines Prepared with Somatic Embryogenesis**	5_MO7_SE
**Muscat Ottonel 14**		6_MO14_SE
**Trilla**		7_T_SE
**Sziren**		8_SZ_SE
**Trilla**	**Lines Prepared with Meristem Culture**	9_T_ME
**Sziren**		10_SZ_ME

**Table 2:** Results of the virus diagnostics of the mother plants.

Library Code	Virus Diagnostics	Viruses						Viroids	
		GFkV	GRVFV	GSyV-1	GRSPaV	GVT	GPGV	HSVd	GYSVd-1
MO7	sRNS HTS	1	1	1	0	0	0	1	1
	RT-PCR	1	1	1	1	0	0	1	1
MO14	sRNS HTS	1	1	1	0	0	1	1	1
	RT-PCR	1	(1)	1	1	1	1	1	1
T	sRNS HTS	0	0	1	0	0	1	1	1
	RT-PCR	0	0	1	1	(1)	1	1	1
SZ	sRNS HTS	0	0	1	0	0	1	1	1
	RT-PCR	0	0	1	1	1	1	1	1

1 indicates the presence of the virus, while 0 shows when the virus was not detected; (1) cases when the presence of the virus was possible.

**Table 3:** Results of the somatic embyogenesis.

Cultivars/Clones	Numbers of Anthers	Numbers of Calli	Numbers of Embryogenic Calli	Numbers of Regenerated Lines	Numbers of in Vitro Lines Tested via sRNA HTS *
Muscat ottonel H-7-3	243	17	5	18	7
Muscat ottonel H-14-1	273	15	2	19	8
‘Trilla’	280	15	9	55	12
’Sziren’	251	34	9	32	11

* Leaves of in vitro grapevine were collected for the small RNA HTS.

**Table 4:** Results of the meristem in vitro cultures.

Cultivar/Clone	Numbers of Meristems	Numbers of Growing Shoots	Numbers of Small Plantlets with no Roots	Numbers of Regenerated Independent Lines Plants with Roots Tested via sRNA HTS *
Muscat Ottonel H-7-3	5	0	0	0
Muscat Ottonel H-14-1	7	0	0	0
’Trilla’	42	15	11	5
’Sziren’	25	10	9	4

* Leaves of in vitro grapevine were collected for the small RNA HTS.

**Table 5:** Summary of the results of the virus elimination efficiency of SE and ME.

Library Code	Virus Diagnostics		Viruses						Viroids	
			GFkV	GRVFV	GSyV-1	GRSPaV	GVT	GPGV	HSVd	GYSVd-1
MO7_SE	sRNS HTS		0	0	0	0	0	0	1	1
	RT-PCR	pool	0	0	0	0	0	0	0	0
		lines	n	N	n	0/7	0	n	0/7	1/7
MO14_SE	sRNS HTS		0	0	0	0	0	0	1	1
	RT-PCR	pool	0	0	0	0	(1)	0	0	1
		lines	n	n	n	0/8	0/8	n	0/8	2/8
T_SE	sRNS HTS		0	0	0	0	0	0	1	1
	RT-PCR	pool	0	0	0	0	0	0	1	1
		lines	n	n	n	0/12	0/12	0/12	2/12	11/12
T_ME	sRNS HTS		0	0	0	1	0	0	1	1
	RT-PCR	pool	0	0	0	(1)	0	0	1	1
		lines	n	n	n	3/5	(1)/5	(2)/5	5/5	5/5
SZ_SE	sRNS HTS		0	0	0	1	0	0	1	1
	RT-PCR	pool	0	0	0	1	0	0	1	0
		lines	n	n	n	6/11	0/11	0/11	2/11	2/11
SZ_ME	sRNS HTS		0	0	0	1	1	1	1	(1)
	RT-PCR	pool	0	0	0	1	1	1	1	0
		lines	n	n	n	2/4	2/4	2/4	2/4	0/4

The presence of viruses was tested by sRNA HTS or using virus-specific RT-PCR. With the latter method, pools of RNA (and, where necessary, individual lines) were tested. The number 0 indicates that the test was done but that the virus was not detected; n is present when individual lines were not tested for the presence of the virus; 1 indicates the presence of the virus. For the individual lines that were tested, the numbers indicate in how many lines the virus was detected. The green colour shows when the elimination was successful, while orange shows when it was not. Light green indicates that the elimination was successful but not in all of the resulting sanitized lines or when the small RNA HTS and RT-PCR gave different results.

## Supplementary Materials

The following are available online: https://www.mdpi.com/2223-7747/9/12/1782/s1. Supplementary Figures for Turcsan et al: Figure S1: Validation of small RNA HTS by RT-PCR for the presence of regulated viruses using virus specific primers., Figure S2: Virus diagnostics of the seven independent lines generated with somatic embryogenesis from Muscat Ottonel clone 7 by RT-PCR., Figure S3: Virus diagnostics of the eight independent lines generated with somatic embryogenesis from Muscat Ottonel clone 14 by RT-PCR., Figure S4: Virus diagnostics of the twelve independent lines generated with somatic embryogenesis (SE) or meristem culture (ME) from ‘Trilla’ by RT-PCR., Figure S5: Virus diagnostics of the eleven independent lines generated with somatic embryogenesis (SE) or meristem culture (ME) from ‘Sziren’ by RT-PCR and Supplementary Tables for Turcsan et al: Table S1: Review of published somatic embryogenesis based virus elimination protocols and their efficiency on grapevine., Table S2: Basic information of the samples used for small RNA sequencing library preparation., Table S3: Initial statistics of the sequenced small RNA libraries., Table S4: Detailed results of the bioinformatics analysis of small RNA HTS of different libraries., Table S5: Sequences of the PCR primers used for virus detection with their appropriate references.
