# Supplementary material for: HTS-Based Monitoring of the Efficiency of Somatic Embryogenesis and Meristem Cultures Used for Virus Elimination in Grapevine
Source: Plants (Basel). 2020 Dec 16;9(12):1782. doi: 10.3390/plants9121782 (PMC7765609; doi:10.3390/plants9121782)
Supplement: Supplementary file 1 [file plants-09-01782-s001.zip › Supplementary Figures for Turcsan et al.docx]

Supplementary Figures to

HTS-based monitoring of the efficiency of somatic embryogenesis and meristem cultures used for virus elimination in grapevine

Mihaly Turcsan^.1^, Emese Demian^.2^, Tunde Varga^.2^, Nikoletta Jaksa-Czotter^.2^, Erno Szegedi^.1^, Robert Olah^.1^ and Eva Varallyay^.2^*

***** Correspondence: [varallyay.eva@abc.naik.hu](mailto:varallyay.eva@abc.naik.hu)

**
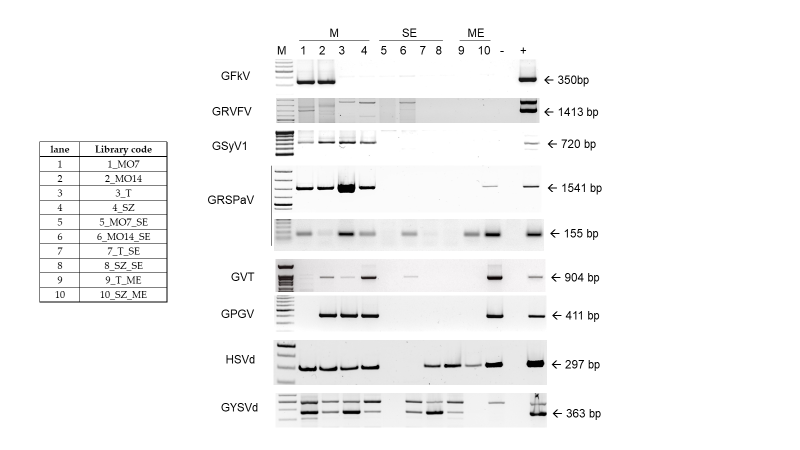
**

**Figure S1.** **Validation of small RNA HTS by RT-PCR for the presence of regulated viruses using virus specific primers.** M-GeneRuler 100bp Plus, -/+ negative and positive controls using water or cDNA prepared from a virus infected plant. Primers which was used to amplify the particular virus are listed in Table S5. M are samples from the mother plants, while SE and ME are the independent lines which were virus eliminated by somatic embryogenesis and meristem cultures, respectively. The lines correspond to the sequenced small RNA libraries as is shown in the small Table on the left side.





**Figure S2.** **Virus diagnostics of the seven independent lines generated with somatic embryogenesis from Muscat Ottonel clone 7 by RT-PCR.** M-GeneRuler 100bp Plus, -/+ negative and positive controls using water or cDNA prepared from a virus infected plant. Primers which was used to amplify the particular virus are listed in Table S5.





**Figure S3.** **Virus diagnostics of the eight independent lines generated with somatic embryogenesis from Muscat Ottonel clone 14 by RT-PCR.** M-GeneRuler 100bp Plus, -/+ negative and positive controls using water or cDNA prepared from a virus infected plant. Primers which was used to amplify the particular virus are listed in Table S5.





**Figure S4.** **Virus diagnostics of the t****welve independent lines generated with somatic embryogenesis (SE) or meristem culture (ME) from ‘Trilla’ by RT-PCR.** M-GeneRuler 100bp Plus, -/+ negative and positive controls using water or cDNA prepared from a virus infected plant. Primers which was used to amplify the particular virus are listed in Table S5.





**Figure S5.** **Virus diagnostics of the** **eleven independent lines generated with somatic embryogenesis (SE) or meristem culture (ME) from ‘Sziren’ by RT-PCR**. M-GeneRuler 100bp Plus, -/+ negative and positive controls using water or cDNA prepared from a virus infected plant. Primers which was used to amplify the particular virus are listed in Table S5.
